# An optimized small animal tumour model for experimentation with low energy protons

**DOI:** 10.1371/journal.pone.0177428

**Published:** 2017-05-18

**Authors:** Elke Beyreuther, Kerstin Brüchner, Mechthild Krause, Margret Schmidt, Rita Szabo, Jörg Pawelke

**Affiliations:** 1 Helmholtz-Zentrum Dresden – Rossendorf, Dresden, Germany; 2 OncoRay – National Center for Radiation Research in Oncology, Faculty of Medicine and University Hospital Carl Gustav Carus, Technische Universität Dresden, Helmholtz-Zentrum Dresden - Rossendorf, Dresden, Germany; 3 Technische Universität Dresden, Germany; 4 Department of Radiotherapy and Radiation Oncology, Faculty of Medicine and University Hospital Carl Gustav Carus, Technische Universität Dresden, Dresden, Germany; 5 German Cancer Consortium (DKTK), partner site Dresden, and German Cancer Research Center (DKFZ), Heidelberg, Germany; 6 National Center for Tumor Diseases (NCT), partner site Dresden, Germany; 7 Attosecond Light Pulse Source, ELI-HU Nonprofit Ltd., Szeged, Hungary; ENEA Centro Ricerche Casaccia, ITALY

## Abstract

**Background:**

The long-term aim of developing laser based particle acceleration towards clinical application requires not only substantial technological progress, but also the radiobiological characterization of the resulting ultra-short and ultra-intensive particle beam pulses. After comprehensive cell studies a mouse ear tumour model was established allowing for the penetration of low energy protons (~20 MeV) currently available at laser driven accelerators. The model was successfully applied for a first tumour growth delay study with laser driven electrons, whereby the need of improvements crop out.

**Methods:**

To optimise the mouse ear tumour model with respect to a stable, high take rate and a lower number of secondary tumours, Matrigel was introduced for tumour cell injection. Different concentrations of two human tumour cell lines (FaDu, LN229) and Matrigel were evaluated for stable tumour growth and fulfilling the allocation criteria for irradiation experiments. The originally applied cell injection with PBS was performed for comparison and to assess the long-term stability of the model. Finally, the optimum suspension of cells and Matrigel was applied to determine applicable dose ranges for tumour growth delay studies by 200 kV X-ray irradiation.

**Results:**

Both human tumour models showed a high take rate and exponential tumour growth starting at a volume of ~10 mm^3^. As disclosed by immunofluorescence analysis these small tumours already interact with the surrounding tissue and activate endothelial cells to form vessels. The formation of delimited, solid tumours at irradiation size was shown by standard H&E staining and a realistic dose range for inducing tumour growth delay without permanent tumour control was obtained for both tumour entities.

**Conclusion:**

The already established mouse ear tumour model was successfully upgraded now providing stable tumour growth with high take rate for two tumour entities (HNSCC, glioblastoma) that are of interest for future irradiation experiments at experimental accelerators.

## 1. Introduction

Current state-of-the-art treatment of cancer patients most often includes radiotherapy with high energy photons or electrons that are delivered by compact clinical electron linear accelerators. Besides, a small but increasing number of patients are treated with ions (protons and heavier ions) [1] that are characterized by an inverse depth-dose profile with a dose maximum (Bragg peak) and a steep distal fall-off at the end of their range. Advantageously, the Bragg peak position depends on particle energy allowing to deposit a maximum dose within the tumour under sparring of the normal tissue. However, owing to their much higher mass the acceleration of ions to clinically relevant energies requires large accelerators, whose facility size, including magnetic beam transport systems with rotating gantries for multi-directional irradiation of patients, and costs limit the widespread distribution of ion therapy.

As one potential alternative the acceleration of ions on micrometre scale by high-intensity laser systems was investigated during the last decade [2]. The necessary technological developments of laser accelerators and its appertaining equipment, like beam transport and dose delivery systems for clinical use is an ongoing process. Moreover, the clinical implementation of this new technique also demands for extensive radiobiological studies in order to characterize the resulting ultra-short and ultra-intensive (pulse dose rate ~10^10^ Gy/min) particle beam pulses [2,3]. Up to now, several in vitro studies with laser driven particles have been performed [4–8] preparing in vivo studies as the next step in the translational chain [8].

The recently established mouse ear tumour model [9] provides subcutaneous tumours that are small enough to be penetrated by low energy protons (E ~20 MeV) currently available for radiobiological studies at laser driven accelerators [8]. To verify this model under realistic and challenging conditions at an experimental laser accelerator, a full scale irradiation experiment was performed comparing the tumour growth delay after treatment with laser driven and clinical LINAC electrons [10]. Although the model was established for proton treatment, electron irradiation was chosen, since the technological progress of the laser accelerators did not yet allow for stable and reproducible proton irradiation at that time. This previous experiment identified several drawbacks of the model. A general one is a high rate of ~20% of fast growing secondary tumours during follow up that necessitate sacrificing of animals before the primary tumour reaches its final size. Another drawback was the reduced and varying take rate of 60–90%. Unlike the establishment at a laboratory 200 kV X-ray tube [9], studies at the low number of (prototype) laser accelerator systems are characterized by very limited beam access and by beam loss due to the lower reliability of laser accelerators at the present development stage. To deal with these conditions, a higher number of animals compared to the experiment at the clinical LINAC was scheduled to this experimental arm. To encounter longer times of beam loss at the laser accelerator and to handle the large total number of animals injected and irradiated at two different cities, the number of animals to be irradiated at the same day was reduced by spreading the tumour cell injections in a staggered manner over a few weeks [10]. However, the actually lower and unstable take rate still reduced the number of evaluable animals and hampered the planning of future experiments.

In the present study, the mouse ear tumour model should be improved by applying Matrigel (MG) for tumour cell injection. MG supports and stabilises the tumour take without changing the histological appearance [11] and should therefore help to reduce heterogeneity and cell outflow to increase the number of animals in analysis. In a first step, the model was optimised for the head and neck squamous cell carcinoma model FaDu, for which it was originally established [10]. Subsequently, the injection parameters obtained for FaDu were used to adapt the model to the glioblastoma LN229 that was introduced as a second tumour entity of interest for proton treatment. After optimisation, a suitable dose range for future tumour growth delay studies was experimentally determined for both tumour entities.

## 2. Methods

### 2.1 The mouse ear tumour model

Experiments were performed using 7-to-14-week-old female and male NMRI (nu/nu) mice continuously purchased from the breeding facility at Oncoray—National Center for Radiation Research in Oncology in Dresden. The animal facility and the experiments were approved according to the German animal welfare regulations and to the local ethics committee (Approval 24–9168.11-1/2013-44, Saxony State Directorate, Dresden, Germany). Animal housing provided 12 h light– 12 h dark cycle, constant temperature of 26–27°C and relative humidity of 45–60% with maximal 10 mice per cage of Euro Standard Type III. Mice were fed with commercial laboratory animal diet for nude mice and water *ad libitum*. To further suppress their immune response, mice were whole-body irradiated with 4 Gy (200 kV X-rays, 20 mA, ~1 Gy/min) three days before cell injection. Anaesthesia was generally performed by 10 mg/kg Xylazin (i.p., Rompun, Bayer Health Care GmbH, Germany) and 100 mg/kg Ketamin (Ketamin 500 Curamed, Curamed Pharma, Germany)

Tumour growth studies were carried out with the undifferentiated human squamous cell carcinoma (HNSCC) FaDu [12,13] and the human glioblastoma (GBM) cell line LN229 ([14]. Cell lines were maintained in DMEM with 4.5 g/l stable glutamine (Biochrom, Germany) containing 10% FCS (Sigma-Aldrich, Germany), 1% Penicillin/Streptomycin (Biochrom) and 1% sodium pyruvate (Gibco, Germany). Additionally, FaDu medium was supplemented with 20 mM HEPES and 1% 100x non-essential amino acids (all Biochrom), that of LN229 with 7.5% sodium carbonate (Gibco). All cell cultures were maintained at 37°C and 5% CO_2_ in humidified atmosphere.

Subcutaneous tumour growing was induced by injecting 25–30 μl of the different tumour cell suspensions in the middle of the right ears of anaesthetised mice [9,15] by using an insulin syringe. For the FaDu model, tumour growth was explored in 162 mice after inoculation of 1*10^6^ FaDu cells resolved in PBS [10] or of 1*10^3^–1*10^6^ cells in pure (9.7 mg/ml) or diluted MG (1:1 dilution of Matrigel Basement Membrane Matrix, BD Biosciences, USA with PBS, Biochrom). The growth of LN229 tumours was surveyed for suspensions of 1*10^2^–1*10^5^ cells in pure MG and of 1*10^5^ cells in PBS again scheduling 18 mice per group, 90 mice altogether. Tumour growth was continuously checked by calliper measurements and corresponding tumour volumes were calculated by the formula of a rotational ellipsoid π/6 x a x b^2^, where a is the longest and b the shorter tumour axis perpendicular to a. Tumour origin was routinely checked by microsatellite analysis and standard H&E staining.

### 2.2 Setup for 200 kV X-ray irradiation and experiment design

The 200 kV X-ray tube including filters, dosimetry and setup for mouse irradiation are described in detail by Brüchner et al. [9]. Briefly, a collimator (6 mm Pb + 2 mm Al) with four openings of 6 mm in diameter was installed at the X-ray tube allowing for simultaneous irradiation of ear tumours of up to four animals protecting their bodies against radiation. Anaesthetised mice were positioned in mouse setup boxes [16] with the tumour bearing ears fixed on sidewise attached PMMA blocks and were placed horizontally under the collimator superimposing the fixed ears and the collimator openings. The positioning of the tumour within a circular grove marking the irradiation field of 5 mm in diameter and the control thereof was described in Schürer et al. [16]. Referring to the setup used at the laser accelerator, a tuneable heating system was also included in the X-ray tube setup that indirectly heat up the mouse setup box to ensure that the anaesthetized mice can keep their body temperature.

The dose homogeneity within the irradiation field and the dose rate of 0.975 Gy/min at tumour position were determined with GafChromic EBT-2 and EBT-3 films (ISP Corp., New York, USA) calibrated for 200 kV X-rays against two Farmer ionization chambers (type 30010, PTW, Germany) that were readout with a Unidos electrometer (PTW).

The optimum concentrations of cells and MG (see 3.1) determined in the first part of the experiments were used for injection in those animals (138 per entity) scheduled for irradiation experiments and histological studies. Again, the animals were continuously checked for tumour growth firstly by eye and after reaching a size of around 2 mm by calliper measurement. When the tumours reached a diameter of 2.5–3.5 mm (*a* axes) and fulfil the allocation criteria (see 3.4) the animals were randomly allocated to the untreated control and the different treatment groups (FaDu: 0, 3.8, 7.9, 11.9, 15.9 Gy; LN229: 0, 3.5, 7, 10.5, 14 Gy). The dose values were specified in accordance to preceding experiments with FaDu tumours on mouse ear (unpublished); whereas for LN229 similar radiosensitivity and comparable dose values were expected from tumour control studies. Mice of the “0 Gy” groups were anaesthetised and positioned in mouse setup boxes, like those of the other dose groups, but sham irradiated.

### 2.3 Histological studies

To confirm that well perfused and well-defined tumours were included in the experiments, 30 randomly chosen animals of each entity were sacrificed and their tumours were extracted and immediately frozen in liquid nitrogen, when tumour diameters of 1–4 mm were achieved. The tumour microenvironment was analysed on 10 μm tumour sections using the hypoxia marker pimonidazole (Hydroxyprobe Omni Kit, Natural Pharmacia Int., Burlington, MA, USA), the perfusion marker Hoechst 33342 (Sigma Aldrich) and CD31 as marker for the vascular endothelium (cluster of differentiation 31, BD). The staining and scanning processes of tumour sections have been described before [9, 17]; post-processing of fluorescence images was performed with the free software Fiji [18]. Histology of tumours and of adjacent tissues was characterized by standard H&E staining using an Axio Imager M1 microscope (Zeiss, Germany) and the software AxioVision (Version SE64, Rel 4.9.1, Zeiss).

### 2.4 Follow up and analysis of tumour growth data

Tumour diameters were measured two to three times a week during the follow ups of 120 days for FaDu and of 180 days for LN229, respectively. The animals were sacrificed when the tumour reached the diameter of 7–8 mm, at the end of follow up or when they appeared to suffer. To assess the different injection media and cell concentrations, the tumour take rates, potential allocation rates and allocation times were compared. Tumour volume doubling times (VDT) were calculated in the range of exponential tumour growth.

For the 200 kV X-ray irradiation experiment relative tumour volumes were calculated with respect to the tumour volume at allocation day and the times required to achieve a certain relative volume were averaged for all animals of an experimental group. The radiation induced tumour growth delay (GD_V5_) was calculated by subtracting the average time the untreated control tumours need to achieve the fivefold relative tumour volume from the average time required by irradiated tumours of the particular dose groups. Animals showing permanent local tumour control during follow up or developing nearby secondary tumours were excluded from analysis.

Statistical analysis by Student’s t-test with Welch-correction and Kolmogorow—Smirnow test to proof normal distribution was performed with GraphPad Prism (Vers. 4.0, GraphPad Software, USA) considering values of p < 0.05 as statistically significant. Graphical representation of take rates and tumour growth curves was realised by the software OriginPro 8G (OriginLab Corporation, Northampton, USA).

## 3. Results and discussion

### 3.1 Take rate studies and general tumour growth parameter

**Fig 1:**
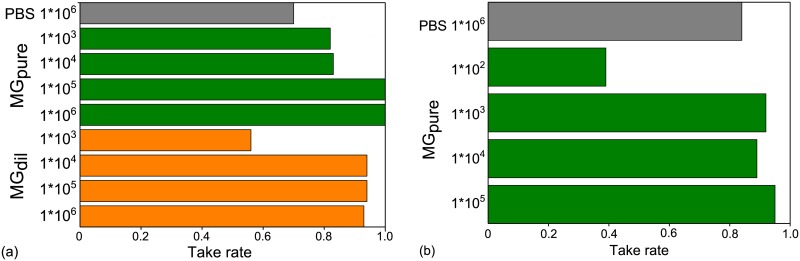
Tumour take rates for both entities. Tumour take rates for (a) HNSCC cell line FaDu and (b) human GBM cell line LN229 after injection of varying cell concentrations in PBS, undiluted MG (MG_pure_) or a 1:1 dilution of MG and PBS (MG_dil_). Depicted are the merged results of two and three injections of LN229 and FaDu, respectively, temporally separated by 4–8 weeks.

Starting with the HNSCC FaDu, the tumour growth after injection of different suspensions of MG and tumour cells in the right mouse ear was evaluated to find the optimum mixture of cells and MG that result in a high take rate and stable tumour growth. The results of this optimization process are resumed in Fig 1a showing in general a higher take rate after inoculation of FaDu tumour cells in combination with MG, either pure or diluted, than after the sole application of cells in PBS. Moreover, focussing on the injection of 105–10^6^ cells in undiluted MG the high take rate of almost ~100% was stable over three staggered injections, which is a noticeable improvement compared to the variable take rate of 60–90% found before [10]. Although this growth promoting effect of MG was optimistically awaited for the mouse ear tumour model, it can not be generalized. For another FaDu model, where the tumour cells were injected in the flanks of nude mice, no impact of MG on tumour take rate and general growth parameters was found [19]. Referring to the results for the FaDu carcinoma, the injections of LN229 glioblastoma cells were just performed with pure MG and one PBS group for comparison. Again, stable and high take rates of ~ 90% were obtained by injecting at least 1000 cells with MG (Fig 1b); whereas the injection of fewer cells or with PBS result in a lower take rate.

**Fig 2:**
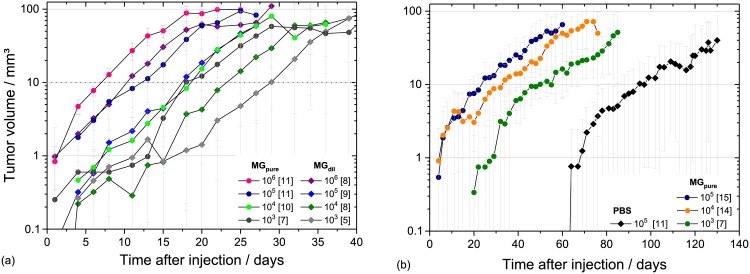
Tumour growth curves on mouse ear. Tumour growth curves obtained for (a) FaDu and (b) LN229 for the different cell/MG suspensions. The measured tumour volumes (see S1 Table for FaDu and S2 Table for LN229) in dependence on time were averaged for all animals of the corresponding group with the number of animals per group given in brackets. Grey dotted horizontal lines indicate the starting volumes of 5 mm^3^ and 10 mm^3^ for the exponential tumour growth of FaDu and LN229, respectively.

As shown in Fig 2, the different injection media also influence the time required to start exponential tumour growth, which most often marks the start of experiments. In line with other studies [11] tumour growth was faster when more concentrated MG was used and when more cells were injected; the extremum was the injection of LN229 with PBS where a remarkable shift of more than one month was observed (Fig 2b). Despite these shifts in time, general growth parameters, like VDT and minimum volume for exponential tumour growth were not altered by injection media and cell concentration. VDT of 2–4 days comparable to those of the previous study [10] and to data of FaDu xenografts on mice legs [20] were measured for all experimental groups of the HNSCC FaDu. In the same manner, consistent VDT of 9–12 days were obtained for all groups of LN229 tumours. The constant volume of 5–10 mm^3^ (Fig 2) required to start the exponential growth might be a general parameter of the mouse ear tumour model.

### 3.2 Long-term stability of the FaDu mouse ear model

**Fig 3:**
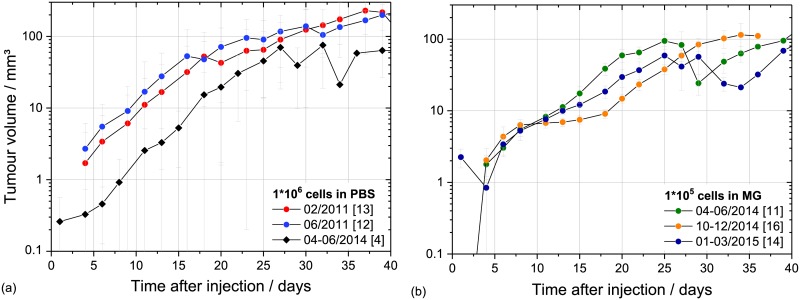
Long-time stability of FaDu mouse ear model. Long-term stability of the FaDu mouse ear model shown in the averaged tumour growth curves measured after inoculation of 1*10^6^ cells in PBS (a) and of 1*10^5^ cells in MG (b) for the different experiment campaigns. The number of animals is given in brackets; the error bars represent the SEM.

With respect to long-time in vivo studies the stability of the mouse ear tumour model was investigated by comparing FaDu tumour growth characteristics obtained after inoculation of 1*10^6^ cells in PBS for several independent experiment campaigns over time (Fig 3a and S3a Table). The average tumour take rate of 70% of the present study is in the broad range of 60–90% found in previous experiments (unpublished, [10]). Moreover, the tumour growth curves of the temporally separated experiment campaigns were very similar, as indicated by a stable VDT of 2–4 days, but showing a shift in time (Fig 3a). Possible reasons for this shift are the application of FaDu cells from a different stock and an altered injection volume. Previously, more care was taken to really inject the intended volume of 50 μl [10]. But, as this “huge” volume was retrospectively assumed as one potential reason for the drop of tumours to the ear base, the injection volume was reduced in the present study to 25–30 μl. This could result in a reduced number of injected cells and hence in an initial tumour growth retardation. Nevertheless, the time shift was compensated by using MG for injection even with a lower number of injected cells (Fig 3b and S3b Table). Considering the growth curves for the different cohorts injected with 1*10^5^ cells in MG (Fig 3b) a plateau phase that lead again to a temporal shift of the exponential growth phase was emerged for the second cohort (10-12/2014). Most probably this altered behaviour was caused by the batch of cells, since for the 2015 cohort no such plateau or shift was observed. However, to circumvent any problems from starting potential treatment too early the minimum volume required for treatment was subsequently increased from 5 mm^3^ to ~ 10 mm^3^.

**Fig 4:**
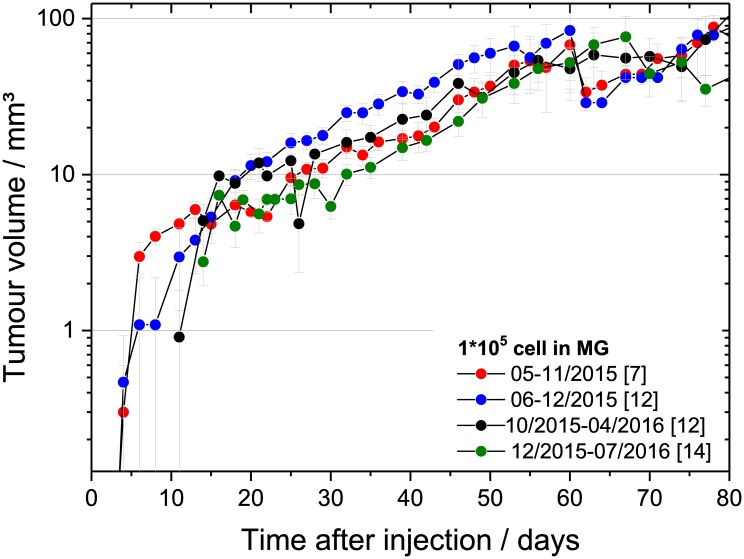
Temporal stability of LN229 growth curves. Average growth curves of LN229 tumours measured after inoculation of 1*10^5^ cells in MG for four temporally different cohorts. The corresponding numbers of animals per cohort are given brackets; the error bars represent the SEM.

The stability of the LN229 mouse ear tumour model could just be studied for the different campaigns of the present experiment (about one year, Fig 4 and S4 Table) showing comparable growth curves and VDT. The long-term stability will be investigated in upcoming experiments.

### 3.3 Histological evaluation of the small mouse ear tumours

**Fig 5:**
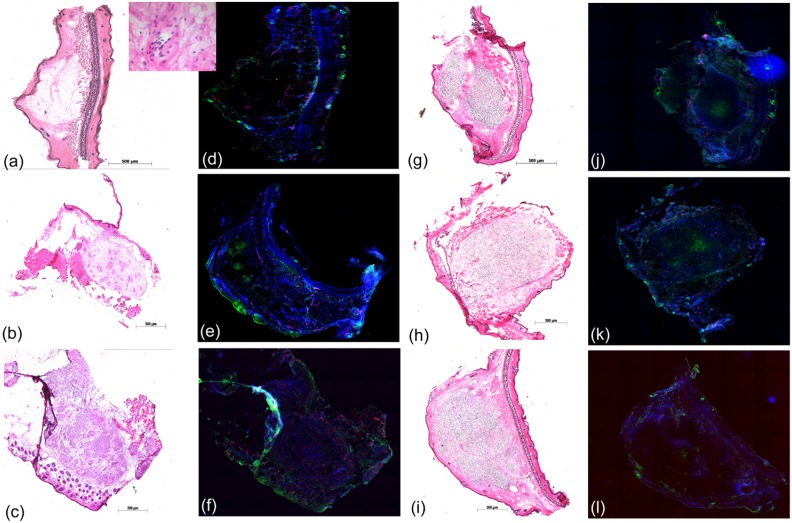
Histology of the small mouse ear tumours. Exemplary tumour sections used to characterize the tumour micromileu for tumour sizes of 1–2 mm (1^st^ line), ≤ 2.5 mm (2^nd^ line) and at treatment size of 3–4 mm (3^rd^ line) for HNSCC FaDu and GBM LN229. The tumour appearance within the surrounding normal tissue was confirmed by classical H&E staining (a-c, g-i) and is shown in 50x magnification, except the inset of Fig 5a, where a magnification of 400x was applied. The analysis of tumour histology takes place by immunofluorescence staining of perfused areas (blue, Hoechst 33342), of small vessels (red, CD31) and of hypoxic regions (green, pimonidazole). Due to technical reasons sections of two different tumours are shown in Fig 5b and 5e, whereas the stained sections of all other pairs were taken from the same tumour.

The experimental application of the mouse ear tumour model, e.g. for irradiation studies, requires solid, circumscribed and vital tumours. To ensure that the chosen starting point fulfil these requirements, the tumour growth was histologically and immunohistochemically investigated for tumour volumes of 2.5–15 mm^3^. For both, HNSCC FaDu and GBM LN229, the tumours at irradiation size appear to be well bordered and growing subcutaneously without infiltration of the skin (Fig 5c and 5i). Applying immunofluorescence analysis (Fig 5f and 5l) evenly distributed perfused areas and small hypoxic regions, but no necrotic areas, were revealed.

The exemplary tumour sections in Fig 5 depict the typical development of xenograft tumours from cell injection to solid tumour, whereas the different growth velocities of the two entities result in slight differences in the individual growth phases (Fig 5a–5c and 5g–5i). Focusing on FaDu, the tumour formation starts with single cells and proceeds with tumour cell clusters in MG at tumour diameters of 1–2 mm and up to 2.5 mm, respectively (Fig 5a and 5b). These clusters are connected in the following, forming a solid tumour at irradiation size of ~3 mm (Fig 5c). Regarding the accompanied immunofluorescence pictures (Fig 5d–5f) the multiplication of vital tumour cells, the sprout of small vessels and the formation of evenly distributed perfused areas within the tumour volume could be followed.

In contrast to FaDu, the slow growing of LN229 tumours provides more time for MG degradation and for solid tumour formation at comparable tumour diameters. As shown in Fig 5g, already at the small dimension of ~1.5 mm a tiny solid tumour with very few MG residues was developed. With increasing size, a small hypoxic area is formed in the middle of the tumour volume (Fig 5k), which in turn stimulate the sprout of small vessels (Fig 5l) and a better perfusion of the tumour.

Finally, for both tumour entities a small solid tumour interspersed with mouse stroma and suitable for treatment was developed at tumour diameters of about 3 mm. Particularly for FaDu, the histological finding of tumour clusters at smaller tumour volumes substantiate the decision to increase the minimum volume that mark the start of experiments to 10 mm^3^. Relative to previous experiments with the mouse ear tumour model, where a start volume of about 5 mm^3^ was applied [9,10], the application and time consuming degradation of MG demand for an increase of the start volume to assure the treatment of vital, solid tumours.

### 3.4 Tumour parameters required to start irradiation experiments

Preparation of in vivo studies at experimental accelerators demand not only for a tumour model with stable take rate and growth parameters, but also for a high proportion of tumours that fulfil the following criteria for allocating an animal into the experiment within a certain period of time:

- Tumour volume of ~10 mm^3^ and dimensions of ~3 mm x 2.5 mm (*a* x *b*)
- Reddish/red tumour indicating sufficient perfusion and a vital tumour
- Position in the middle of mouse ear and uniform shape, ideally round or elliptic
- One single tumour in the target area, no doublets or second tumour on or close to the ear

Applying the above mentioned criteria, the different suspensions for tumour cell injection were assessed with respect to the subsequent (see 3.5.), but also upcoming irradiation experiments (Table 1). As already shown in Fig 2a, the time span between injection and allocation (allocation time) was inversely correlated to the cell number and MG concentration used for tumour cell injection, whereas no such dependency was seen for the allocation rate, i.e. the number of allocated animals relative to the scheduled ones. However, as the allocation rate greatly depends on geometrical facts the experience of the experimentalist in tumour cell injection might have a greater influence than the composition of the tumour cell suspension. This increasing experience was also seen during the present experiment, where the rate of failed injections, for example by accidental piercing of the ear, decreases from 13/162 animals in the case of FaDu to 3/89 for LN229.

**Table 1:** General tumour growth parameters for the different groups. The average allocation time is given in days after injection required to achieve a tumour volume of about 10 mm^3^; the number of animals per group (*n*) and take rates are included for completeness. The differences between the scheduled (18) and actual number of animals per group arose from failed injections, i.e. by accidental ear piercing, premature dead after injection or problems with the cell suspension in the case of the FaDu PBS group.

Cell Number	FaDu				LN229			
	n	Take rate	Allocation rate	Allocation time / d (± sem)	n	Take rate	Allocation rate	Allocation time / d (± sem)
*PBS*								
1*10^6^	10	0.70	0.57	18.8 ± 2.8				
1*10^5^					19	0.84	0.69	106.2 ± 9.7
*9*.*7 mg/ml Matrigel*								
1*10^6^	16	1.0	0.69	7.4 ± 1.5				
1*10^5^	16	1.0	0.69	13.3 ± 1.7	19	0.95	0.50	23.6 ± 2.0
1*10^4^	16	0.81	0.62	19.4 ± 2.0	19	0.89	0.75	28.5 ± 2.7
1*10^3^	17	0.82	0.36	26.0 ± 3.0	13^§^	0.92	0.36	45.8 ± 3.9
1*10^2^					18	0.39	0.0	---
*4*.*85 mg/ml Matrigel (MG*:*PBS 1*:*1)*								
1*10^6^	15	0.93	0.21	13.7 ± 4.6				
1*10^5^	16	0.94	0.53	18.9 ± 1.1				
1*10^4^	16	0.94	0.53	24.9 ± 2.3				
1*10^3^	16	0.56	0.44	27.8 ± 1.7				

^§^ During injection of the 2^nd^ cohort of LN229 premature gelatinisation of MG causes a stop of injection in that particular group; the remaining animals of that group were allotted to the other groups.

To sum up, the injection of 10^5^ cells in undiluted MG was specified as optimum for subsequent radiobiological studies, since for HNSCC FaDu and for LN229 glioblastoma a high and stable take rate and an acceptable allocation time linked with a good allocation rate could be achieved. Of course, the take rate obtained after injection of 10^6^ FaDu tumour cells was also very high, but the very fast tumour growing of this group demand for prompt treatment and argues against its application at experimental accelerators where a delay cannot be excluded. Beyond take rate, MG was also introduced to reduce outflow of cells and with it the growth of secondary tumours. However, since the tumours were not treated during the optimisation process, the follow up was rather short and related to this a low rate of secondary tumour induction was seen. To be more realistic, the secondary tumour incidence for the two tumour models was therefore assessed during the irradiation campaign.

### 3.5. Dose range determination for radiobiological tumour growth delay studies

The application of any tumour model for radiobiological tumour growth delay studies require the definition of a reasonable dose range that result in a significant growth delay compared to untreated tumours, but not in permanent tumour control. To study the dose dependent radiation response FaDu and LN229 tumour cells were each inoculated in 108 animals, from which 95–100% developed a tumour confirming the optimised injection parameters and the stability of the model. Accordingly, allocation times of 14.5 ± 0.8 days and of 23.4 ± 1.0 days, similar to that measured before, were found for FaDu and LN229, respectively. The corresponding allocation rates vary between 74% for FaDu and 89% for LN229 being noticeable higher than in the optimisation experiments (Table 1) and the rate of 42–52% obtained previously for FaDu [10]. However, the high allocation rates should not be overrated since it remains unclear if the application of MG, the batch of cells or the increasing experience of the experimenters, e.g. in cell injection, might be responsible for it.

Despite of these improvements one drawback of the FaDu mouse ear tumour model, namely the high rate of secondary tumours, was not abolished after optimisation. Like in the previous study of Oppelt et al. [10], where PBS was used for injection, about one fifth of the allocated animals developed a secondary tumour close to the head at the base of the right ear. The FaDu origin of these secondary tumours was histologically confirmed, whereas the genesis could not be resolved definitely. Most often secondary tumours at the ear base appear not before four weeks after injection, whereas the rarer appearance of earlier, simultaneous tumours was probably directly caused by injection and resulted in the exclusion of these animals from experiment. A clear correlation to irradiation could not resolved in the present experiment, since the majority of tumours grown during the optimization process (see 3.4) and those of the unirradiated control groups (incl. sham irradiated ones) reached their final size within four weeks after injection. As in some cases secondary tumours also appeared in these groups one can speculate if a longer follow up will result in higher incidence rates. Anyhow, due to better blood supply secondary tumours appearing at the ear base grow faster than the primary ones, especially after high dose irradiation that is correlated to longer growth delay, and some of the animals have to be sacrificed before the follow up of the primary tumour was finished. For doses up to 7.9 Gy the endpoint “time to fivefold relative tumour volume” was most often reached. Nevertheless, also subtracting those animals that were excluded for other reasons, like general health condition, about 70% of the animals allocated to the FaDu experiment are evaluable, whereas a higher proportion of 89% could be evaluated for LN229. For this particular model, merely 8% of the animals develop a secondary tumour, which moreover grew very slowly and seldom disturb the evaluation of the primary ones. In the other way, LN229 mouse ear tumours tend to scab more often lowering the statistic for the sevenfold or tenfold relative volume increase. The determination of GD_V5_ in the present study was however not affected by this phenomenon. Taking all facts together, drop-outs occurred at all steps from inoculation to analysis, esp. for FaDu, but due to the high take rate the total number of animals to be scheduled for growth delay experiments could still be reduced.

**Fig 6:**
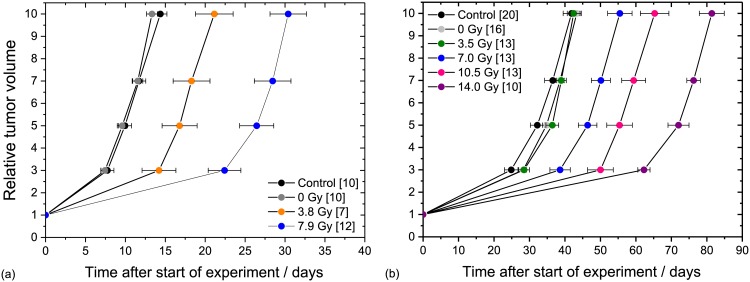
200 kV X-ray dose dependent tumour growth curves. Dose dependent tumour growth curves for the mouse ear tumour models of HNSCC FaDu (a) and the glioblastoma LN229 (b). The average times (± SEM) to achieve a certain relative volume increase are shown for all animals in the group; the number of animals per group are given in brackets.

Comparing the tumour growth curves of the untreated controls and the sham irradiated groups, a significant difference was neither found for FaDu (Fig 6a) nor for LN229 tumours (Fig 6b), indicating that the handling procedure at 200 kV X-ray treatment, i.e. anaesthesia, positioning and maintenance in the mouse setup boxes, has no influence on tumour growth. This result is in line with previous findings of KHT mouse sarcoma [9] and HNSCC FaDu [10] on mouse ear and indicates that the presently introduced injection of MG has also no effect. For FaDu GD_V5_ of 6.8 ± 2.3 days and of 16.5 ± 2.3 days were measured after irradiation with 3.8 Gy and 7.9 Gy (Fig 6a), respectively. For higher doses no growth curves were calculated since 60–80% of the tumours were controlled over the whole follow-up. Relative to FaDu the LN229 tumour model seems to be slightly more radioresistant as indicated by not significantly different growth curves of the control and the 3.5 Gy group (Fig 6b) and a low local control rate of one animal in each of the dose groups of 10.5 Gy and 14 Gy. One possible reason for this higher radioresistance might be the hypoxic tumour region that appears already at small tumour volumes for the LN229 tumours (Fig 5j–5l). The applicable dose range for tumour growth delay studies seems to be wider for LN229 tumours. But, the doses should be defined carefully as the tumour growth curves obtained in the present work after irradiation with doses of 7 Gy and 10.5 Gy are so close that the distinction of GD_V5_ is at the limit of significance (p = 0.056), whereas the GD_V3_ and GD_V7_ are significantly different. The GD_V5_ of FaDu and LN229 are summarized in Table 2; the tumour growth data for both tumour entities, i.e. the times required to achieve the corresponding relative tumour volumes, are summarized in S5 Table.

**Table 2:** GD_V5_ values for the two tumour models. For LN229 treated with a dose of 3.5 Gy the value was not calculated since the corresponding time values are not significantly different from control. Dose errors include systematic and measuring uncertainties of film calibration and dose measurement; errors of the individual GD_V5_ values were calculated by error propagation.

HNSCC FaDu		GBM LN229	
Dose ± ΔD / Gy	GD_V5_ ± se / d	Dose ± ΔD / Gy	GD_V5_ ± se / d
3.8 ± 0.2 Gy	6.8 ± 2.3	3.5 ± 0.2 Gy	---
7.9 ± 0.5 Gy	16.5 ± 2.3	7.0 ± 0.4 Gy	14.2 ± 3.3
11.9 ± 0.7 Gy	---	10.5 ± 0.6 Gy	23.2 ± 4.1
15.9 ± 1.0 Gy	---	14.0 ± 0.8 Gy	39.8 ± 3.6

### 3.6 Limitations of the model

The present study reveals not only the optimised injection parameters that result in a high take rate and in stable tumour growth, but also some aspects that should be taken into account by using the model for future experiments. The first aspect is the injection of tumour cells, which should be done by a trained person to assure a low failure rate, i.e. by accidental ear piercing, and there with a high allocation rate.

The second aspect is the definition of doses for irradiation experiments or drug testing, which should be considered carefully, since for FaDu the risk of secondary tumours and for LN229 the tendency of tumour scabbing will increase for longer follow up times, respectively. Moreover, the application of the mouse ear tumour model for tumour growth delay studies is limited to maximum doses of 8 Gy for FaDu and ~14 Gy for LN229. Above these values the determination of tumour control instead of growth delay should be applied for radiobiological characterisation.

### Conclusion

The mouse ear tumour model was considerably improved for FaDu by the application of MG instead of PBS and by an optimised, reduced number of 10^5^ cells for tumour cell injection. The increased and now stable take rate of > 95% allows the application of a lower number of animals by retaining the same statistical significance. A constant allocation time and high allocation rate enable the scheduling of irradiation campaigns at experimental accelerators with limited beam time and reduced beam reliability (i.e. increased beam loss). Moreover, it could be shown that the FaDu tumour model was stable over more than one year, which is one essential prerequisite for long-term irradiation experiments and experiment replication. For the LN229 tumour model the stability of tumour growth parameters was already confirmed over months and will be further proven in upcoming experiments.

Beside these radiation independent properties of the model, a reasonable dose range for future radiobiological growth delay studies was determined for both the HNSCC FaDu and the GBM LN229. Taking into account the different relative biological effectiveness between 200 kV X-rays used for the dose definition and any other radiation quality, the model is now prepared, e.g. for the study at laser driven proton beams, but also for other applications with low penetrating radiations like the investigation of micro beam treatment [21]. The main parameters of the mouse ear model for both tumour entities are summarized in Table 3 in order to facilitate the selection of the most convenient entity for a particular application.

**Table 3:** Selection table for future irradiation experiments. The summarized parameters shoulp help to decide which tumour entity is appropriate for the particular experiment. The secondary tumours were most often found a few weeks after injection at the ear base and were histologically confirmed to be of the same origin as the primary tumours, whereas the genesis could not be resolved definitely (see section 3.5).

	FaDu	LN229
*After injection*		
Take rate	> 95%	
Projectable start of treatment	14.5 ± 0.8 days	23.4 ± 1.0 days*
*During experiment*		
Allocation rate	74%	89%
VDT	2–4 days	9–12 days
Beam loss or delay	Time shift problematic	Shift of 1 day possible
Recommended dose range	< 8 Gy	< 14 Gy
*After experiment campaign*		
Follow up	120 days	180 days
Secondary tumours	~ 20%, fast growing	~ 8%, slowly growing

*Sometimes difficult to define, since LN229 tumours will be reddish, not red.

## Supporting information

S1 Table HNSCC FaDu tumour growth after injection. Tumour volumes (Vol) and corresponding sem (standard error of the mean) in dependence on time (days) after inoculation of HNSCC FaDu tumour cells in the right mouse ear. For injection the tumor cells were suspended either in pure (9.7 mg/ml) or in diluted (4.85 mg/ml, MG:PBS 1:1) Matrigel. The number of animals per group is given in brackets.
(DOCX)

S2 Table Growth of LN229 glioblastoma tumours. Group mean tumour volumes (Vol) and standard error of the means (sem) in dependence on time (days) after injection of LN229 tumour cells in the right mouse ear. The tumor cells were suspended in pure (9.7 mg/ml) Matrigel and the number of animals per group is given in brackets.
(DOCX)

S3 Table HNSCC FaDu tumour growth over time. Long-term stability of the FaDu mouse ear model shown by the averaged tumour growth curves measured as respective tumour volume increase (± sem) after inoculation (a) of 1*10^6^ cells in PBS and (b) of 1*10^5^ cells in Matrigel for the different temporally separated experiment campaigns. The number of animals is given in brackets.
(DOCX)

S4 Table Temporal stability of LN229 growth curves. Average tumour volumes (± SEM) measured for LN229 tumours after inoculation of 1*10^5^ cells in MG for four temporally different cohorts. The corresponding numbers of animals per cohort are given brackets.
(DOCX)

S5 Table 200 kV X-ray dose dependent tumour growth curves. Dose dependent tumour growth data for the mouse ear tumour models of HNSCC FaDu (a) and the glioblastoma LN229 (b). The average times (± SEM) to achieve a certain relative volume increase are given as mean values over all animals in the group; the number of animals per group are given in brackets.
(DOCX)

## Abbreviations

CD31: cluster of differentiation 31
DMEM: Dulbecco´s Modified Eagle Medium
FCS: foetal calf serum
H&E: haematoxylin and eosin
HNSCC: head and neck squamous carcinoma
i.p.: intraperitoneally
i.v.: intravenous
PBS: phosphate buffered saline
SEM: standard error of the mean
VDT: volume doubling time(s)
