# Supplementary material for: An optimized small animal tumour model for experimentation with low energy protons
Source: PLoS One. 2017 May 18;12(5):e0177428. doi: 10.1371/journal.pone.0177428 (PMC5436688; doi:10.1371/journal.pone.0177428)
Supplement: S1 Table — Tumour volumes (Vol) and corresponding sem (standard error of the mean) in dependence on time (days) after inoculation of HNSCC FaDu tumour cells in the right mouse ear. For injection the tumor cells were suspended either in pure (9.7 mg/ml) or in diluted (4.85 mg/ml, MG:PBS 1:1) Matrigel. The number of animals per group is given in brackets. (DOCX) [file pone.0177428.s001.docx]

**S1: HNSCC FaDu tumour growth after injection**; Tumour volumes (Vol) and corresponding sem (standard error of the mean) in dependence on time (days) after inoculation of HNSCC FaDu tumour cells in the right mouse ear. For injection the tumor cells were suspended either in pure (9.7 mg/ml) or in diluted (4.85 mg/ml, MG:PBS 1:1) Matrigel. The number of animals per group is given in brackets.

|  | | | | |  |  |  |  |  |  |  |  |  |  |  |  |
| --- | --- | --- | --- | --- | --- | --- | --- | --- | --- | --- | --- | --- | --- | --- | --- | --- |
| **Days after injection** | **MG diluted [8] 1*10^6 cells** | | **MG diluted [9] 1*10^5 cells** | | **MG diluted [8] 1*10^4 cells** | | **MG diluted [5] 1*10^3 cells** | | **MG pure [11] 1*10^6 cells** | | **MG pure [11] 1*10^5 cells** | | **MG pure [10] 1*10^4 cells** | | **MG diluted [7] 1*10^3 cells** | |
|  | **Vol /mm³** | **sem** | **Vol/ mm³** | **sem** | **Vol/ mm³** | **sem** | **Vol/ mm³** | **sem** | **Vol/ mm³** | **sem** | **Vol/ mm³** | **sem** | **Vol/ mm³** | **sem** | **Vol/ mm³** | **sem** |
| 1 | 0.98 | 1.45 | 0.06 | 0.17 | 0.00 | 0.00 | 0.00 | 0.00 | 0.83 | 0.49 | 0.00 | 0.00 | 0.00 | 0.00 | 0.25 | 0.67 |
| 4 | 1.99 | 2.21 | 0.32 | 0.31 | 0.22 | 0.63 | 0.26 | 0.37 | 4.71 | 1.10 | 1.78 | 1.92 | 0.47 | 0.73 | 0.60 | 1.59 |
| 6 | 3.26 | 3.16 | 0.57 | 0.58 | 0.32 | 0.65 | 0.46 | 0.77 | 7.76 | 1.16 | 3.04 | 2.48 | 0.69 | 0.81 | 0.60 | 1.59 |
| 8 | 5.01 | 4.13 | 1.51 | 1.62 | 0.48 | 0.63 | 0.71 | 0.97 | 12.80 | 2.86 | 5.48 | 3.78 | 1.22 | 1.07 | 0.60 | 1.59 |
| 11 | 12.27 | 10.29 | 2.18 | 2.43 | 0.29 | 0.63 | 0.94 | 1.29 | 27.17 | 7.03 | 8.27 | 4.98 | 1.62 | 1.75 | 0.74 | 1.97 |
| 13 | 18.06 | 17.53 | 4.04 | 5.94 | 0.75 | 0.88 | 1.68 | 2.29 | 43.20 | 11.34 | 11.28 | 7.95 | 2.76 | 2.63 | 0.97 | 2.36 |
| 15 | 30.38 | 29.41 | 4.43 | 3.85 | 0.82 | 1.00 | 0.84 | 1.87 | 50.64 | 10.00 | 17.45 | 13.57 | 4.56 | 3.23 | 3.25 | 6.29 |
| 18 | 52.31 | 48.37 | 11.96 | 11.78 | 3.71 | 3.48 | 1.19 | 1.84 | 88.47 | 20.06 | 38.63 | 24.86 | 8.32 | 5.80 | 10.17 | 17.08 |
| 20 | 62.15 | 65.36 | 18.57 | 19.73 | 4.27 | 3.49 | 1.41 | 1.83 | 86.83 | 21.16 | 59.36 | 40.57 | 15.33 | 11.81 | 12.22 | 16.97 |
| 22 |  |  | 27.25 | 25.93 | 7.85 | 6.21 | 3.04 | 2.58 | 98.88 | 23.49 | 65.18 | 37.05 | 28.12 | 21.55 | 17.67 | 23.76 |
| 25 |  |  | 46.56 | 47.87 | 14.29 | 13.83 | 4.77 | 2.28 |  |  | 94.44 | 61.79 | 44.34 | 31.52 | 31.66 | 32.20 |
| 27 |  |  | 59.01 | 71.78 | 22.12 | 19.36 | 7.15 | 4.10 |  |  |  |  | 59.09 | 37.28 | 43.04 | 47.39 |
| 29 |  |  |  |  | 29.35 | 29.22 | 10.12 | 5.89 |  |  |  |  | 80.22 | 58.18 | 58.15 | 60.65 |
| 32 |  |  |  |  | 59.97 | 61.05 | 20.84 | 17.10 |  |  |  |  |  |  |  |  |
| 34 |  |  |  |  |  |  | 35.57 | 23.27 |  |  |  |  |  |  |  |  |
| 36 |  |  |  |  |  |  | 49.62 | 26.78 |  |  |  |  |  |  |  |  |
| 39 |  |  |  |  |  |  | 74.66 | 14.78 |  |  |  |  |  |  |  |  |
| 41 |  |  |  |  |  |  | 93.90 | 1.96 |  |  |  |  |  |  |  |  |
| 43 |  |  |  |  |  |  | 137.87 | 8.37 |  |  |  |  |  |  |  |  |
| 46 |  |  |  |  |  |  | 205.25 | 0.00 |  |  |  |  |  |  |  |  |
