# Supplementary material for: An optimized small animal tumour model for experimentation with low energy protons
Source: PLoS One. 2017 May 18;12(5):e0177428. doi: 10.1371/journal.pone.0177428 (PMC5436688; doi:10.1371/journal.pone.0177428)
Supplement: S2 Table — Group mean tumour volumes (Vol) and standard error of the means (sem) in dependence on time (days) after injection of LN229 tumour cells in the right mouse ear. The tumor cells were suspended in pure (9.7 mg/ml) Matrigel and the number of animals per group is given in brackets. (DOCX) [file pone.0177428.s002.docx]

| **S2:** **Growth of LN229 glioblastoma tumours**. Group mean tumour volumes (Vol) and standard error of the means (sem) in dependence on time (days) after injection of LN229 tumour cells in the right mouse ear. The tumor cells were suspended in pure (9.7 mg/ml) Matrigel and the number of animals per group is given in brackets. | | | | | | | | |
| --- | --- | --- | --- | --- | --- | --- | --- | --- |
| **Days after injection** | **PBS [11] 1*10^5 cells** | | **MG pure [15] 1*10^5 cells** | | **MG pure [14] 1*10^4 cells** | | **MG diluted [7] 1*10^3 cells** | |
|  | **Vol /mm³** | **sem** | **Vol /mm³** | **sem** | **Vol /mm³** | **sem** | **Vol /mm³** | **sem** |
| 1 | 0.00 | 0.00 | 0.00 | 0.00 | 0.00 | 0.00 | 0.00 | 0.00 |
| 4 | 0.00 | 0.00 | 0.54 | 1.32 | 0.91 | 1.62 |  |  |
| 6 | 0.00 | 0.00 | 1.87 | 3.14 | 2.03 | 2.21 |  |  |
| 8 | 0.00 | 0.00 | 2.59 | 3.24 | 2.55 | 2.38 |  |  |
| 11 | 0.00 | 0.00 | 3.48 | 4.07 | 4.33 | 3.72 |  |  |
| 13 | 0.00 | 0.00 | 3.64 | 3.65 | 4.26 | 3.60 |  |  |
| 15 | 0.00 | 0.00 | 4.41 | 3.44 | 3.14 | 3.21 |  |  |
| 18 | 0.00 | 0.00 | 7.41 | 4.25 | 3.62 | 4.31 |  |  |
| 20 | 0.00 | 0.00 | 7.54 | 5.51 | 3.05 | 3.28 |  |  |
| 22 | 0.00 | 0.00 | 8.41 | 5.36 | 4.07 | 3.60 | 0.75 | 1.98 |
| 25 | 0.00 | 0.00 | 12.25 | 7.77 | 6.28 | 4.39 | 0.75 | 1.98 |
| 27 | 0.00 | 0.00 | 12.42 | 5.47 | 7.72 | 5.06 | 0.90 | 2.37 |
| 29 | 0.00 | 0.00 | 13.20 | 6.70 | 8.75 | 4.57 | 1.05 | 2.77 |
| 32 | 0.00 | 0.00 | 18.38 | 7.94 | 9.08 | 5.36 | 3.13 | 4.46 |
| 34 | 0.00 | 0.00 | 18.25 | 12.35 | 11.52 | 7.22 | 2.90 | 3.97 |
| 36 | 0.00 | 0.00 | 21.34 | 13.82 | 12.74 | 7.91 | 4.03 | 4.32 |
| 39 | 0.00 | 0.00 | 25.29 | 16.89 | 13.93 | 9.55 | 6.04 | 3.08 |
| 41 | 0.00 | 0.00 | 23.37 | 14.31 | 14.16 | 8.93 | 6.37 | 3.47 |
| 43 | 0.00 | 0.00 | 29.05 | 16.66 | 16.42 | 10.30 | 7.72 | 4.26 |
| 46 | 0.00 | 0.00 | 38.76 | 24.21 | 20.33 | 15.10 | 9.44 | 3.08 |
| 48 | 0.00 | 0.00 | 41.07 | 26.40 | 19.37 | 11.84 | 9.36 | 3.06 |
| 50 | 0.00 | 0.00 | 44.95 | 29.01 | 22.69 | 16.28 | 10.06 | 3.68 |
| 53 | 0.00 | 0.00 | 53.46 | 37.75 | 33.40 | 26.58 | 11.03 | 3.27 |
| 55 | 0.00 | 0.00 | 50.30 | 35.50 | 38.21 | 28.33 | 9.89 | 2.40 |
| 57 | 0.00 | 0.00 | 53.69 | 36.92 | 44.50 | 37.04 | 14.62 | 9.18 |
| 60 | 0.00 | 0.00 | 65.51 | 53.19 | 49.93 | 43.24 | 16.25 | 9.88 |
| 62 | 0.00 | 0.00 |  |  | 47.04 | 26.25 | 14.32 | 6.34 |
| 64 | 0.76 | 1.69 |  |  | 53.98 | 31.18 | 18.95 | 9.58 |
| 67 | 0.76 | 1.69 |  |  | 56.75 | 32.32 | 19.75 | 10.44 |
| 69 | 1.24 | 2.14 |  |  | 62.35 | 37.33 | 21.19 | 9.26 |
| 71 | 2.21 | 2.71 |  |  | 72.13 | 42.19 | 21.65 | 9.64 |
| 74 | 2.89 | 2.86 |  |  |  |  | 23.03 | 10.42 |
| 76 | 3.71 | 3.91 |  |  |  |  | 25.44 | 15.70 |
| 78 | 4.39 | 4.48 |  |  |  |  | 28.62 | 16.11 |
| 81 | 4.73 | 5.50 |  |  |  |  | 36.00 | 22.38 |
| 83 | 4.63 | 4.69 |  |  |  |  | 46.10 | 44.97 |
| 85 | 5.10 | 5.11 |  |  |  |  | 51.41 | 45.95 |
| 88 | 6.93 | 6.52 |  |  |  |  |  |  |
| 90 | 7.52 | 7.62 |  |  |  |  |  |  |
| 92 | 7.97 | 8.00 |  |  |  |  |  |  |
| 95 | 10.27 | 10.35 |  |  |  |  |  |  |
| 97 | 9.94 | 9.21 |  |  |  |  |  |  |
| 99 | 12.39 | 11.65 |  |  |  |  |  |  |
| 102 | 12.24 | 11.52 |  |  |  |  |  |  |
| 104 | 17.36 | 16.86 |  |  |  |  |  |  |
| 106 | 17.17 | 15.95 |  |  |  |  |  |  |
| 109 | 20.59 | 18.27 |  |  |  |  |  |  |
| 111 | 19.06 | 17.39 |  |  |  |  |  |  |
| 113 | 17.73 | 18.04 |  |  |  |  |  |  |
| 116 | 15.68 | 17.09 |  |  |  |  |  |  |
| 118 | 17.46 | 27.32 |  |  |  |  |  |  |
| 119 | 24.72 | 23.84 |  |  |  |  |  |  |
| 120 | 24.63 | 40.33 |  |  |  |  |  |  |
| 123 | 30.06 | 33.46 |  |  |  |  |  |  |
| 125 | 28.98 | 41.79 |  |  |  |  |  |  |
| 126 | 37.41 | 35.73 |  |  |  |  |  |  |
| 127 | 29.13 | 41.73 |  |  |  |  |  |  |
| 130 | 40.05 | 46.32 |  |  |  |  |  |  |
