# Supplementary material for: An optimized small animal tumour model for experimentation with low energy protons
Source: PLoS One. 2017 May 18;12(5):e0177428. doi: 10.1371/journal.pone.0177428 (PMC5436688; doi:10.1371/journal.pone.0177428)
Supplement: S3 Table — Long-term stability of the FaDu mouse ear model shown by the averaged tumour growth curves measured as respective tumour volume increase (± sem) after inoculation (a) of 1*106 cells in PBS and (b) of 1*105 cells in Matrigel for the different temporally separated experiment campaigns. The number of animals is given in brackets. (DOCX) [file pone.0177428.s003.docx]

| **3a) HNSCC FaDu: tumor growth after injection of 1*10^6 cells in PBS** | | | | | | |  |  |
| --- | --- | --- | --- | --- | --- | --- | --- | --- |
| **Days after injection** | **02/2011 [13]** | | **Days after injection** | **06/2011 [12]** | | **Days after injection** | **04-06/2014 [4]** | |
|  | **Vol /mm³** | **sem** |  | **Vol /mm³** | **sem** |  | **Vol /mm³** | **sem** |
| 4 | 1.7 | 2.9 | 3 | 2.7 | 3.4 | 1 | 0.26 | 0.30 |
| 6 | 3.4 | 4.4 | 5 | 5.5 | 5.7 | 4 | 0.33 | 0.39 |
| 9 | 6.1 | 9.1 | 7 | 9.1 | 10.8 | 6 | 0.46 | 0.33 |
| 11 | 11.1 | 14.5 | 10 | 16.9 | 27.1 | 8 | 0.92 | 1.02 |
| 13 | 16.7 | 23.6 | 12 | 27.7 | 30.8 | 11 | 2.56 | 2.55 |
| 16 | 31.8 | 41.4 | 14 | 53 | 70.3 | 13 | 3.29 | 1.91 |
| 18 | 52.5 | 61.5 | 17 | 47.8 | 37.2 | 15 | 5.27 | 3.78 |
| 20 | 42.8 | 41.2 | 19 | 71.3 | 59.5 | 18 | 15.32 | 16.28 |
| 23 | 63.1 | 62.9 | 21 | 95.4 | 78 | 20 | 19.57 | 17.86 |
| 25 | 65.1 | 51 | 24 | 90.3 | 76.4 | 22 | 30.48 | 24.02 |
| 27 | 90.2 | 67.5 | 26 | 117.1 | 80.6 | 25 | 45.43 | 32.04 |
| 30 | 124.1 | 113.9 | 28 | 138.6 | 101.3 | 27 | 70.10 | 51.68 |
| 32 | 143.2 | 92.8 | 31 | 105 | 47.9 | 29 | 39.37 | 29.55 |
| 34 | 173.1 | 122.3 | 33 | 134.9 | 57.3 | 32 | 75.40 | 56.55 |
| 37 | 229 | 172.3 | 35 | 167.8 | 61.7 | 34 | 21.21 | 0 |
| 39 | 216.8 | 190.3 | 38 | 198.8 | 78.6 | 36 | 58.32 | 0 |
| 41 | 113.5 | 103.1 | 40 | 223.5 | 105.8 | 39 | 63.62 | 0 |
| 44 | 151.6 | 109.7 | 42 | 267.3 | 143.1 | 41 | 63.62 | 0 |
| 46 | 176 | 110.7 |  |  |  | 43 | 78.54 | 0 |
| 48 | 148.4 | 80.3 |  |  |  | 46 | 122.52 | 0 |
| 51 | 216 | 176 |  |  |  |  |  |  |
| 53 | 260.9 | 200.9 |  |  |  |  |  |  |
| 55 | 318.3 | 0 |  |  |  |  |  |  |

**S3: HNSCC FaDu tumour growth over time.** Long-term stability of the FaDu mouse ear model shown by the averaged tumour growth curves measured as respective tumour volume increase (± sem) after inoculation (a) of 1*10^6^ cells in PBS and (b) of 1*10^5^ cells in Matrigel for the different temporally separated experiment campaigns. The number of animals is given in brackets.

| **3b) HNSCC FaDu: tumor growth after injection of 1*10^5 cells in MG** | | | | | | |  |  |
| --- | --- | --- | --- | --- | --- | --- | --- | --- |
| **Days after injection** | **04-06/2014 [11]** | | **Days after injection** | **10-12/2014 [16]** | | **Days after injection** | **01-03/2015 [14]** | |
|  | **Vol /mm³** | **sd** |  | **Vol /mm³** | **sd** |  | **Vol /mm³** | **sd** |
| 1 | 0 | 0 | 1 | 6.00E-04 | 2.45E-04 | 1 | 2.2516 | 0.66526 |
| 4 | 1.78455 | 0.5792 | 4 | 2.04 | 0.94106 | 4 | 0.84 | 0.84 |
| 6 | 3.03545 | 0.7483 | 6 | 4.36 | 1.57404 | 6 | 3.4 | 0.99197 |
| 8 | 5.48182 | 1.1385 | 8 | 6.32 | 1.13772 | 8 | 5.26 | 1.46137 |
| 11 | 8.27 | 1.5003 | 11 | 6.74 | 1.01272 | 11 | 7.6 | 1.28957 |
| 13 | 11.27545 | 2.398 | 13 | 6.94 | 0.90089 | 13 | 10 | 0.92952 |
| 15 | 17.44636 | 4.0901 | 15 | 7.48 | 0.96819 | 15 | 12.12 | 0.65757 |
| 18 | 38.62819 | 7.497 | 18 | 9.06 | 1.23394 | 18 | 18.52 | 3.59157 |
| 20 | 59.36364 | 12.233 | 20 | 14.74 | 1.93148 | 20 | 29.62 | 8.30225 |
| 22 | 65.18111 | 12.35 | 22 | 23.24 | 4.2932 | 22 | 36.9 | 10.9881 |
| 25 | 94.44286 | 23.355 | 25 | 37.82 | 5.82807 | 25 | 59.025 | 19.53127 |
| 27 | 82.99001 | 43.031 | 27 | 59.04 | 10.78298 | 27 | 41.4 | 22.0754 |
| 29 | 24.28 | 7.79 | 29 | 84.14 | 16.85531 | 29 | 56.73333 | 35.1144 |
| 32 | 48.565 | 15.055 | 32 | 102.2667 | 28.23481 | 32 | 23.9 | 5.1 |
| 34 | 63.125 | 15.415 | 34 | 114.75 | 51.15 | 34 | 21.2 | 0 |
| 36 | 78.535 | 6.545 | 36 | 110.9 | 0 | 36 | 32.1 | 0 |
| 39 | 95.03 | 0 |  | -- | -- | 39 | 68.9 | 0 |
| 41 | 143.79 | 0 |  | -- | -- | 41 | 91.6 | 0 |
| -- | -- | -- |  |  |  | 43 | 98.2 | 0 |
