# Supplementary material for: An optimized small animal tumour model for experimentation with low energy protons
Source: PLoS One. 2017 May 18;12(5):e0177428. doi: 10.1371/journal.pone.0177428 (PMC5436688; doi:10.1371/journal.pone.0177428)
Supplement: S4 Table — Average tumour volumes (± SEM) measured for LN229 tumours after inoculation of 1*105 cells in MG for four temporally different cohorts. The corresponding numbers of animals per cohort are given brackets. (DOCX) [file pone.0177428.s004.docx]

**S4: Temporal stability of LN229 growth curves.** Average tumour volumes (± SEM) measured for LN229 tumours after inoculation of 1*10^5^ cells in MG for four temporally different cohorts. The corresponding numbers of animals per cohort are given brackets.

| **Days after injection** | **05-11/2015 [7]** | | **Days after injection** | **06-12/2015 [12]** | | **Days after injection** | **10/2015 - 04/2016 [12]** | | **Days after injection** | **12/2015-07/2016 [14]** | |
| --- | --- | --- | --- | --- | --- | --- | --- | --- | --- | --- | --- |
|  | **Vol /mm³** | **sd** |  | **Vol /mm³** | **sd** |  | **Vol /mm³** | **sd** |  | **Vol /mm³** | **sd** |
| 1 | 8.33E-04 | 1.67E-04 | 1 | 1.11E-04 | 1.11E-04 | 1 | 0 | 0 | 1 | 0 | 0 |
| 4 | 0.30 | 0.30 | 4 | 0.47 | 0.47 | 4 | 0 | 0 | 4 | 0 | 0 |
| 6 | 2.98 | 0.68 | 6 | 1.09 | 1.09 | 7 | 0 | 0 | 7 | 0 | 0 |
| 8 | 4.02 | 0.47 | 8 | 1.09 | 1.09 | 11 | 0.91 | 0.91 | 11 | 0 | 0 |
| 11 | 4.83 | 0.47 | 11 | 2.97 | 1.62 | 14 | 5.07 | 1.37 | 14 | 2.76 | 0.83 |
| 13 | 5.97 | 0.73 | 13 | 3.80 | 1.47 | 16 | 9.82 | 0.00 | 16 | 7.38 | 1.02 |
| 15 | 4.82 | 0.64 | 15 | 5.34 | 1.73 | 18 | 8.79 | 2.05 | 18 | 4.67 | 1.26 |
| 18 | 6.38 | 0.66 | 18 | 9.16 | 1.42 | 21 | 11.88 | 2.84 | 19 | 6.90 | 1.00 |
| 20 | 5.77 | 0.36 | 20 | 11.44 | 2.10 | 22 | 9.82 | 0.00 | 21 | 5.60 | 1.38 |
| 22 | 5.38 | 0.34 | 22 | 12.10 | 1.81 | 25 | 12.28 | 2.85 | 22 | 6.93 | 0.63 |
| 25 | 9.57 | 1.03 | 25 | 15.97 | 2.46 | 26 | 4.84 | 2.49 | 23 | 6.93 | 0.63 |
| 27 | 10.82 | 1.14 | 27 | 16.56 | 2.07 | 28 | 13.53 | 3.25 | 25 | 6.99 | 1.40 |
| 29 | 11.00 | 1.46 | 29 | 17.82 | 2.13 | 32 | 16.06 | 3.30 | 26 | 8.63 | 1.17 |
| 32 | 15.02 | 1.37 | 32 | 24.94 | 2.93 | 35 | 17.33 | 3.33 | 28 | 8.74 | 1.72 |
| 34 | 13.35 | 1.88 | 34 | 24.93 | 3.80 | 39 | 22.62 | 5.87 | 30 | 6.25 | 1.05 |
| 36 | 16.22 | 1.99 | 36 | 28.41 | 4.29 | 42 | 24.07 | 7.36 | 32 | 10.08 | 1.59 |
| 39 | 17.02 | 2.22 | 39 | 34.03 | 5.62 | 46 | 38.43 | 16.65 | 35 | 11.15 | 1.73 |
| 41 | 17.74 | 3.94 | 41 | 32.84 | 4.22 | 49 | 31.34 | 8.01 | 39 | 14.89 | 2.52 |
| 43 | 20.20 | 2.80 | 43 | 39.06 | 5.38 | 53 | 45.23 | 12.57 | 42 | 16.57 | 2.57 |
| 46 | 30.18 | 5.10 | 46 | 50.96 | 7.97 | 56 | 54.03 | 14.84 | 46 | 21.94 | 4.45 |
| 48 | 33.80 | 7.70 | 48 | 56.04 | 11.18 | 60 | 47.75 | 17.87 | 49 | 30.87 | 7.50 |
| 50 | 36.94 | 6.10 | 50 | 59.96 | 14.79 | 63 | 58.57 | 22.36 | 53 | 38.46 | 9.93 |
| 53 | 50.35 | 15.17 | 53 | 66.65 | 22.87 | 67 | 55.82 | 15.04 | 56 | 47.86 | 13.36 |
| 55 | 53.58 | 20.48 | 55 | 56.33 | 21.05 | 70 | 57.20 | 17.38 | 60 | 52.29 | 12.24 |
| 57 | 48.63 | 23.50 | 57 | 69.57 | 22.27 | 74 | 49.20 | 19.94 | 63 | 68.14 | 19.42 |
| 60 | 67.90 | 32.54 | 60 | 84.05 | 50.55 | 77 | 73.47 | 30.10 | 67 | 76.53 | 27.02 |
| 62 | 33.80 | 8.10 | 62 | 28.9 | 0 | 81 | 117.80 | 45.15 | 70 | 44.38 | 12.85 |
| 64 | 37.50 | 8.60 | 64 | 28.9 | 0 | 84 | 89.55 | 51.85 | 74 | 52.80 | 23.00 |
| 67 | 44.00 | 6.30 | 67 | 41.9 | 0 | 88 | 37.7 | 0 | 77 | 35.33 | 7.88 |
| 69 | 44.00 | 6.30 | 69 | 41.9 | 0 | 91 | 37.7 | 0 | 81 | 44.07 | 15.17 |
| 71 | 55.40 | 13.50 | 71 | 41.9 | 0 | 95 | 53 | 0 | 84 | 60.20 | 19.71 |
| 74 | 58.05 | 16.15 | 74 | 63.6 | 0 | 98 | 65.4 | 0 | 88 | 44.9 | 9.6 |
| 76 | 70.05 | 28.15 | 76 | 78.5 | 0 | 102 | 72 | 0 | 91 | 83.55 | 14.65 |
| 78 | 88.35 | 16.35 | 78 | 78.5 | 0 | 105 | 72 | 0 | 95 | 68.9 | 0 |
| 81 | 72.00 | 0.00 | 81 | 91.6 | 0 | 109 | 95 | 0 | 98 | 74.2 | 0 |
| 83 | 95.00 | 0.00 | 83 | 131.9 | 0 | 112 | 110.9 | 0 | 102 | 98.2 | 0 |
| 85 | 110.90 | 0.00 | 85 | 141.4 | 0 | 116 | 192.4 | 0 |  |  |  |
| 88 | 165.90 | 0.00 |  |  |  |  |  |  |  |  |  |
