# Supplementary material for: An optimized small animal tumour model for experimentation with low energy protons
Source: PLoS One. 2017 May 18;12(5):e0177428. doi: 10.1371/journal.pone.0177428 (PMC5436688; doi:10.1371/journal.pone.0177428)
Supplement: S5 Table — Dose dependent tumour growth data for the mouse ear tumour models of HNSCC FaDu (a) and the glioblastoma LN229 (b). The average times (± SEM) to achieve a certain relative volume increase are given as mean values over all animals in the group; the number of animals per group are given in brackets. (DOCX) [file pone.0177428.s005.docx]

**S5*:* 200 kV X-ray dose dependent tumour growth curves.** Dose dependent tumour growth data for the mouse ear tumour models of HNSCC FaDu (a) and the glioblastoma LN229 (b). The average times (± SEM) to achieve a certain relative volume increase are given as mean values over all animals in the group; the number of animals per group are given in brackets.

| **a) Dose dependent tumour growth curves for HNSCC FaDu** | | | | |  |  |  |  |  |  |  |  |
| --- | --- | --- | --- | --- | --- | --- | --- | --- | --- | --- | --- | --- |
|  | Time after start of experiment required to achieve the x-fold tumour volume increase | | | | | | | |  |  |  |  |
|  | V3 | | | V5 | | | V7 | | | V10 | | |
| Dose group | Time /d | sem | n | Time /d | sem | n | Time /d | sem | n | Time /d | sem | n |
| Control | 7.75 | 0.81 | 10 | 9.95 | 0.81 | 10 | 11.7 | 0.85 | 10 | 14.38 | 0.82 | 8 |
| 0 Gy | 7.45 | 0.53 | 10 | 9.6 | 0.62 | 10 | 11.55 | 0.63 | 10 | 13.33 | 0.68 | 9 |
| 3.8 Gy | 14.21 | 2.12 | 7 | 16.79 | 2.20 | 7 | 18.29 | 2.30 | 7 | 21.14 | 2.36 | 7 |
| 7.9 Gy | 22.42 | 2.05 | 12 | 26.45 | 2.14 | 11 | 28.45 | 2.29 | 10 | 30.4 | 2.28 | 10 |
|  |  |  |  |  |  |  |  |  |  |  |  |  |
|  |  |  |  |  |  |  |  |  |  |  |  |  |
| **b) Dose dependent tumour growth curves for GBM LN229** | | | | |  |  |  |  |  |  |  |  |
|  | Time after start of experiment required to achieve the x-fold tumour volume increase | | | | | | | |  |  |  |  |
|  | V3 | | | V5 | | | V7 | | | V10 | | |
| Dose group | Time /d | sem | n | Time /d | sem | n | Time /d | sem | n | Time /d | sem | n |
| Control | 24.85 | 1.91 | 20 | 32.2 | 1.96 | 20 | 36.54 | 2.29 | 18 | 41.85 | 2.4 | 17 |
| 0 Gy | 28.25 | 0.89 | 16 | 34.8 | 1.12 | 15 | 38.62 | 1.16 | 13 | 43.3 | 1.3 | 10 |
| 3.5 Gy | 28.46 | 1.50 | 13 | 36.42 | 1.81 | 13 | 38.96 | 1.40 | 12 | 42.45 | 1.8 | 11 |
| 7 Gy | 38.62 | 2.88 | 13 | 46.38 | 2.60 | 13 | 50.12 | 2.67 | 13 | 55.46 | 3.5 | 12 |
| 10.5 Gy | 50 | 3.61 | 13 | 55.38 | 3.60 | 13 | 59.35 | 3.36 | 13 | 65.27 | 4.0 | 11 |
| 14 Gy | 62.25 | 1.72 | 10 | 72 | 2.97 | 5 | 76.25 | 1.91 | 6 | 81.38 | 3.5 | 4 |
